# SSFinder: High Throughput CRISPR-Cas Target Sites Prediction Tool

**DOI:** 10.1155/2014/742482

**Published:** 2014-06-26

**Authors:** Santosh Kumar Upadhyay, Shailesh Sharma

**Affiliations:** National Agri-Food Biotechnology Institute, Department of Biotechnology, Government of India, C-127, Industrial Area, S.A.S. Nagar, Phase 8, Mohali, Punjab 160071, India

## Abstract

Clustered regularly interspaced short palindromic repeats (CRISPR) and CRISPR-associated protein (Cas) system facilitates targeted genome editing in organisms. Despite high demand of this system, finding a reliable tool for the determination of specific target sites in large genomic data remained challenging. Here, we report SSFinder, a python script to perform high throughput detection of specific target sites in large nucleotide datasets. The SSFinder is a user-friendly tool, compatible with Windows, Mac OS, and Linux operating systems, and freely available online.

## 1. Introduction

Genome editing is a very useful technology in the research areas related to the functional genomics. Programmable nucleases like zinc finger nucleases (ZFN) and transcription activator-like effector nucleases (TALEN) are well known tools for targeted genome editing [1, 2]. Similarly, clustered regularly interspaced short palindromic repeats (CRISPR) and CRISPR-associated protein (Cas) system enables genome engineering in animals and plants [3–5]. This is an RNA guided system, which consists of a short guide RNA (gRNA) and a Cas9 protein. The gRNA contains 20 nucleotides targeted DNA sequence (known as spacer) that binds to the complementary strand of the target DNA by base-pairing and 79 nucleotides conserved sequence to form a specific hairpin-like fold. The gRNA interacts with Cas9 protein and forms an active ribonucleoprotein complex. The complex binds to the target sequence by base-pairing and cleaves dsDNA at a specific position [6]. A short conserved motif “NGG” (known as a protospacer adjacent motif: PAM) at 3′ downstream of the target sequence is also reported as necessary for cleavage [7].

The CRISPR-Cas system is simple in design as compared to ZFNs and TALENs and highly effective in large and complex genome like 17 Gb allohexaploid wheat also [5]. Some off-target binding of the system is reported [8] which can be abandoned by selecting the highly specific 12 nucleotide seed sequences in 3′ region of the spacer [4, 9]. Besides dsDNA cleavage, the CRISPR-Cas system has been recently modified for nicking and repression of gene activity [10]. It is reported as an effective tool for activation of gene expression also [11]. Due to these novel features, this system is going to play very important role in genome engineering programs. Therefore, a dedicated tool for determination of specific CRISPR-Cas target sites is an utmost requirement for several research groups.

Although detection of CRISPR-Cas target site is simple, which needs direct analysis of DNA sequences for the presence of specific 23 nucleotides (including “NGG” PAM at 3′ end). A few tools like CRISPR Design (http://www.broadinstitute.org/mpg/crispr_design/, http://crispr.mit.edu/), CRISPR Target (http://bioanalysis.otago.ac.nz/CRISPRTarget/crispr_analysis.html) and ZiFiT Targeter (http://zifit.partners.org/ZiFiT/ChoiceMenu.aspx) are recently reported for the determination of target sites [12]; most of these tools are limited to the analysis of small number or size of sequences and cannot modify according to the users need. Further, these are web based tools that depend upon internet connectivity. Some of the tools like CRISPR design (http://crispr.mit.edu/) are limited to the model genomes only (See Supplementary Table 1 in Supplementary Material available online at http://dx.doi.org/10.1155/2014/742482). Therefore, a simple, easy to edit, and high throughput computational tool is required for the analysis of large datasets on the local machine.

Here, we present SSFinder, a freeware written in python to find specific CRISPR-Cas target sites in limited time on even a personal computer. It is an organism independent freeware available at https://code.google.com/p/ssfinder/ under MIT license.

## 2. Materials and Methods

The SSFinder is a Python script and can execute in Windows, Mac OS, and Linux operating systems. It is a low memory request tool, which enables the finding of specific CRISPR-Cas target sites. It can be installed on both personal computers and parallel computing system commonly known as “cluster” dedicated in genome research. It only needs a compatible version of Python (2.2 or higher version) installed in the system.

**Figure 1:**
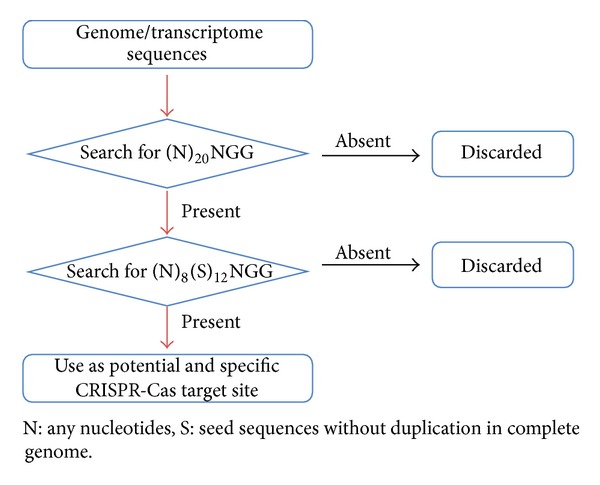
A flow chart showing algorithm of the SSFinder for CRISPR-Cas target site prediction.

A flow chart showing algorithm of the SSFinder is given in Figure 1. The DNA sequence is first analyzed for the occurrence of 23 nucleotide segments (including “NGG” PAM at 3′ end). These segments are further screened for the presence of 12 nucleotide seed sequences, which are distinct in the input sequence data. To further simplify, selected sequences are again classified into four groups on the basis of the start and end nucleotides, which are (1) G/C N_7_S_11_ G/C, (2) G/C N_7_S_11_ A/T, (3) A/T N_7_S_11_ A/T, and (4) A/T N_7_S_11_ G/C (N denotes for any nucleotides and S for seed sequences).

To use SSFinder, users need to download the script or copy-paste the script in a notepad and save as “ssfinder.py” in the directory hosting the Python executable (C:\Python). A working directory containing input file of FASTA formatted nucleotide sequences is also required. Finally, SSFinder can be executed by using the following command line in Linux terminal.

 $python SSFinder.py

**Figure 2:**
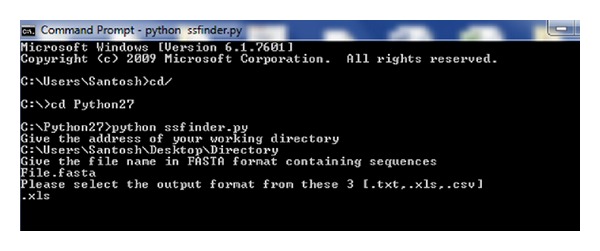
An overview of command prompt for using SSFinder in Windows operating system.

For windows user, an overview for the execution of the SSFinder using command prompt is shown in Figure 2. User needs to provide an address of working directory, file name of input sequences, and desired output format (like  .xls or  .txt or  .csv) when asked by SSFinder. The output file will be saved automatically in the same directory with the file name “SSFinder-output.” In this file, SSFinder provides results in seven distinct columns consisted of (1) identifier of the sequences, (2) classification in four groups based on the start and end nucleotides, (3) potential target sites, including “NGG” PAM sequence, (4) start and (5) end position of target sites, (6) condition with specific 12 nucleotide seed sequences, and (7) specific CRISPR-Cas target sites (Table 1). Sequences from the 7th column can be directly used as spacers in the gRNA designing.

## 3. Results and Discussion

The SSFinder facilitates prediction of CRISPR-Cas target sites in small as well as large genomic data. It is freeware for the researchers and can also be used on personal computers of any configuration. Compatibility with operating systems like Windows, Mac OS, and Linux makes this tool user-friendly for the researcher from nonbioinformatics background as well.

The SSFinder scans a DNA sequence by moving a window of 23 nucleotides at the step size of one nucleotide. Slices with 3′ “NGG” PAM sequence are selected and analyzed for the presence of 12 nucleotide seed sequences. Since the seed sequence determines the specificity of the CRISPR-Cas system [4, 5, 9], the tool ensures that these sequences are not repeated in the entire input genome data [13]. Sequences having distinct seed sequence in entire input sequence file are displayed in output file as specific target sites. Sometimes researchers need to target the genomic region start and end with specific nucleotide (like A/T/G/C). Therefore, the selected slices are further classified into four different motifs to ease the process.

To test the performance of SSFinder, we used two types of large datasets: (1) multiple sequences with cumulative big size (27,416* Arabidopsis thaliana* protein coding genes of ~37 million bases from TAIR10, ftp://ftp.arabidopsis.org/home/tair/Sequences/blast_datasets/TAIR10_blastsets/) and (2) single sequence of large size (X chromosome of ~22 million bases of* Drosophila melanogaster*, accession number NC_004354.3). In the first dataset, a total of 1780846 sites were detected in 27416 genes, in which 1618640 sites in 27239 genes were specific for the CRISPR-Cas binding. The result was in agreement with the earlier report [9]. In case of second dataset, a total of 1058944 sites were detected, in which 977257 were specific. The analyzed data can be available on request. The average speed of the analysis was 34 and 40 kilo bases per minute for first and second dataset, respectively. The average distance between two specific target sites was 22.9 nucleotides in both tested organisms. The results indicated that the SSFinder is a high throughput tool for CRISPR-Cas target site prediction from large datasets in limited time.

## 4. Conclusion

We report SSFinder, a comprehensive tool for the identification of specific CRISPR-Cas target sites with high reliability. It is a freeware, easy to edit, and low memory demand tool compatible with many commonly used operating systems. Our tool is very useful in high throughput inhouse screening applications of large genomes in limited time. This can accelerate the functional genomics research based on the application of CRISPR-Cas system.

## Supplementary Material

We compared the efficiency, excess and flexibility of SSFinder with other reported tools. We found SSFinder is more user friendly and exhaustive tool than others.

## Figures and Tables

**Table 1:** An overview of output file given by SSFinder.

Identifier	Classification	CRISPR-target site with “NGG” PAM	Position		Condition with seed sequences	Specific CRISPR-target site
			Start	End		
Sequence_1	A or TN18A or T	ACTTCTTCGTCCAACTTCTTCGG	6	28	A or TN7S11A or T	ACTTCTTCGTCCAACTTCTT
Sequence_1	A or TN18G or C	TTTGCAAGCCTCATCCATTGTGG	386	408	A or TN7S11G or C	TTTGCAAGCCTCATCCATTG
Sequence_1	G or CN18A or T	CCAAAGTTCTATTTGAGCTAAGG	68	90	G or CN7S11A or T	CCAAAGTTCTATTTGAGCTA
Sequence_1	G or CN18G or C	CTAACCGACCTTCAGCTAACAGG	154	176	G or CN7S11G or C	CTAACCGACCTTCAGCTAAC

## Availability

The SSFinder is a python script, freely available at https://code.google.com/p/ssfinder/.
