# Supplementary material for: SSFinder: High Throughput CRISPR-Cas Target Sites Prediction Tool
Source: Biomed Res Int. 2014 Jun 26;2014:742482. doi: 10.1155/2014/742482 (PMC4095993; doi:10.1155/2014/742482)
Supplement: Supplementary file 1 — We compared the efficiency, excess and flexibility of SSFinder with other reported tools. We found SSFinder is more user friendly and exhaustive tool than others. [file 742482.f1.pdf]

Supplementary table 1. Comparison of the SSFinder with other available tools.

Name of the tool	Link	Input sequence/genome limit	Excess	Adjustability
CRISPR Design tool	http://www.broadinstitute.org/mpg/crispr_design/	1000 bp	Internet dependent	Non-editable
CRISPR Design	http://crispr.mit.edu/	Limited to 15 genome (only 1 from plants)	Internet dependent	Non-editable
CRISPR Target	http://bioanalysis.otago.ac.nz/CRISPRTarget/crispr_analysis.html	Limited to the 50 Mb user database at web interface	Internet dependent	Flexible
ZiFiT Targeter	http://zifit.partners.org/ZiFiT/ChoiceMenu.aspx	Limited to small number of sequences	Internet dependent	Non-editable
SSFinder	http://code.google.com/p/ssfinder	Useful for large genomes	Internet independent	Editable
